# When Silence Is Selective: Self-Silencing Across Relationship Contexts Among Young Adults

**DOI:** 10.3390/bs16060963

**Published:** 2026-06-10

**Authors:** Christin Grothaus

**Affiliations:** Social Science Division, Mahidol University International College, 999 Phutthamonthon 4 Road, Salaya, Nakhon Pathom 73170, Thailand; christinmarie.gro@mahidol.ac.th

**Keywords:** self-silencing, young adults, relationship context, reflexive thematic analysis, Thailand, cultural values

## Abstract

Self-silencing, the suppression of self-expression to preserve relationships, has been theorised as context-dependent, yet research has examined this construct primarily at the between-group level, leaving within-person variation underexplored. This qualitative study examined how self-silencing varies across relationship domains among 53 young adults (30 women, 23 men) at international universities in Thailand, using semi-structured interviews (*n* = 10) and written reflections (*n* = 43) analysed through reflexive thematic analysis. Findings showed that self-silencing was relationship-specific: participants distinguished relationships in which they expressed themselves from those in which they did not, with perceived security as the most consistent distinction. This pattern operated independently of family socialisation: supportive later relationships could undo learned silence, and fragile ones could introduce silence in participants from expressive families. Thai cultural values of *kreng jai* and *jai yen* gave participants a vocabulary for silence but did not dictate where it occurred. The meaning of self-silencing differed by gender, with women more clearly framing silence as care and men as emotional control. Self-silencing carried cumulative psychological costs and took varied behavioural forms. These findings suggest that self-silencing operates as a relationship-specific process rather than a stable personal characteristic.

## 1. Introduction

Why people silence themselves in some relationships, but not in others, remains poorly understood. Self-silencing, the inhibition of self-expression to avoid conflict and preserve close relationships (Jack, 1991), has been associated with a range of adverse outcomes, making this gap consequential. Self-silencing has been linked to depression (Pintea & Gatea, 2021), eating disorders, and reduced wellbeing (Maji & Dixit, 2019), to depressive symptoms in longitudinal studies (Little et al., 2011; Peleg-Sagy & Shahar, 2015), and to intimate partner violence (Inman & London, 2022). In cultural contexts where interpersonal harmony is prioritised, remaining quiet rather than confronting mistreatment may be reinforced by norms in which restraint is seen as a way of protecting relationships (Grothaus, 2024). Jack (1991) developed the self-silencing theory from qualitative interviews with depressed women, proposing that self-silencing arises through internalising culturally prescribed schemas of feminine accommodation, monitored by what she termed the “Over-Eye.” A central but underexplored proposition of the theory is that self-silencing schemas are context-dependent rather than trait-like (Jack & Ali, 2010). How self-silencing differs across a person’s own relationships, and what shapes those differences, remains largely unexamined.

Subsequent research has remained predominantly quantitative (for qualitative exceptions, see Maji & Dixit, 2020; Scott et al., 2023) and has examined self-silencing primarily as a between-person variable, leaving within-person variation across relationships underexplored. The schemas measured by the Silencing the Self Scale (STSS) have been described as “malleable cognitions and behaviors that can change depending on the demands and threats in different contexts” (Jack et al., 2026, p. 3). The original scale validation illustrated this at the between-group level: self-silencing was highest among women in a domestic violence shelter, intermediate among substance-using mothers, and lowest among undergraduates (Jack & Dill, 1992). A narrative review of 126 STSS studies reported that self-silencing tends to be higher in relational contexts marked by conflict, power inequities, and a lack of mutuality, and lower in relationships characterised by satisfaction and mutual support (Jack et al., 2026). Research on relational schemas has similarly proposed that people develop different cognitive representations of different relationships, each containing distinct interpersonal scripts and expectations (Baldwin, 1992), suggesting that self-silencing may vary across a person’s different relationships.

Although Jack’s theory was originally developed in the context of romantic relationships, the STSS has since been adapted for other relational domains, including nursing workplace settings (DeMarco et al., 2007) and school contexts (Patrick et al., 2019). These adaptations implicitly acknowledge that self-silencing may operate across relational domains. How the same individual may self-silence differently across relationship contexts remains an open question. If self-silencing is truly context-dependent, then an individual who self-silences with a romantic partner may communicate openly with family or friends. Understanding where a person self-silences, and what shapes the boundary between expression and silence, would reframe the construct from a characteristic of persons to a characteristic of specific relationships, with implications for how self-silencing is measured, theorised, and addressed clinically.

This reframing also raises a developmental question. Jack (1991) proposed that early relational experiences provide a foundation for self-silencing schemas, but how these earlier patterns relate to later adult relationships has received limited attention. Recent quantitative work has begun to examine this proposition: Iqbal et al. (2025) found that family communication patterns, including conformity orientation, predicted self-silencing among Indian women, while Emran et al. (2023) found that self-silencing mediated the relationship between insecure attachment orientation and depression, with gender moderating this pathway. Whether and how these early patterns shape silence and expression across a person’s different adult relationships has received little attention.

Besides how self-silencing differs across relationships, cultural context has received less attention in the self-silencing literature. Jack et al.’s (2026) review encompassed studies from diverse countries, but none from Thailand or any other nation in Southeast or East Asia. Among the few findings involving Asian populations, Gratch et al. (1995) found that Asian college students reported the highest self-silencing scores among the ethnic groups in their diverse college sample. Research from the Indian subcontinent (Emran et al., 2020, 2023; Iqbal et al., 2025; Maji & Dixit, 2019) and cross-cultural comparisons with Iranian-Australian samples (Davoodi et al., 2025) have begun to explore self-silencing beyond Western contexts, but self-silencing in a Thai or broader Southeast Asian context remains largely unexamined.

This gap is notable because Thai cultural values bear conceptual similarity to self-silencing. *Kreng jai*, often translated as consideration for others’ feelings or reluctance to impose, involves restraining one’s own interests to avoid causing discomfort, conflict, or loss of face (Komin, 1991; Intachakra, 2012). *Jai yen*, or staying calm and cool-hearted, emphasises emotional composure and remaining calm (Rochanavibhata & Marian, 2023). These values shape Thai interpersonal communication, where quietness is considered a virtue (Knutson et al., 2003) and emotional composure during conflict is culturally valued (Komin, 1991). Research on Thai students’ responses to bullying suggests that these values may influence coping strategies, with students remaining quiet rather than confronting mistreatment (Grothaus, 2024). However, *kreng jai* does not appear to have been linked to self-silencing as defined by Jack’s (1991) theory.

Gender has also been examined in the self-silencing literature. Jack’s (1991) original theory focused on women’s self-silencing as a response to feminine gender role socialisation, yet in 19 of 29 gender-comparison studies, men scored significantly higher than women on the STSS, with no study finding women scoring higher (Jack et al., 2026). Research suggests that the motivations underlying self-silencing differ by gender: women tend to frame self-silencing in terms of care and responsibility, while men frame it in terms of emotional control and concealment of vulnerability (Ussher & Perz, 2010). Studies using factor analysis suggest the STSS may not share an identical factor structure across men and women (Cramer & Thoms, 2003), with weaker construct validity in male samples (Remen et al., 2002). These findings suggest that the same STSS scores may reflect different underlying processes across genders, a possibility that qualitative and mixed-gender research is well positioned to explore.

The present study examined self-silencing across relational contexts among culturally diverse young adults at international universities in Thailand. Participants reflected on how they expressed or withheld thoughts and feelings across family, friendship, and romantic relationships, enabling within-person comparison across relational domains. The inclusion of both men and women allowed exploration of gender differences in the motivations and contexts of self-silencing. The cultural context of the sample provided an opportunity to examine how cultural values relate to self-silencing.

Three research questions guided the study. First, how does self-silencing vary across relationship contexts within the same individual? Second, what relational characteristics shape where self-silencing occurs? Third, what role, if any, do cultural values play in participants’ self-silencing patterns?

## 2. Materials and Methods

### 2.1. Participants and Context

Participants were 53 young adults (30 women, 23 men) enrolled at three international universities in Bangkok, Thailand, where instruction is conducted in English. Ages ranged from 18 to 23 years. The sample included Thai (*n* = 31), Thai-Chinese (*n* = 5), Chinese (*n* = 3), Indian (*n* = 3), Myanmar (*n* = 2), American (*n* = 3), and one participant each of Korean, Nepalese, German, Singaporean-Indian, Thai-American, and Thai-New Zealand nationality. Participants were recruited through university networks, including course-based invitations and informal student networks across the three institutions. Participation was voluntary. Convenience sampling was used, as participants self-selected through course enrolment and voluntary response to recruitment invitations. No purposive sampling for particular demographics was employed. The sample was culturally diverse, though Thai and Thai-Chinese participants constituted the majority. The analysis attends to patterns across the full sample, noting where cultural background appears relevant to participants’ accounts. The study received ethical approval from the Institutional Review Board of Mahidol University International College (No. 2020/02-071).

### 2.2. Data Collection

Data were collected from two complementary sources, designed to balance in-depth exploration with broader engagement. First, 10 participants (7 women, 3 men) took part in individual semi-structured interviews lasting 45–75 min. Interviews were conducted in English by the author and trained research assistants, who also supported participant recruitment and initial contact. Their involvement helped create a more accessible and comfortable context for participation, particularly given the author’s role as a university lecturer. Interview questions explored communication patterns across family, friendship, and romantic relationships; experiences of disagreement, conflict, unfair treatment, and aggressive or controlling behaviour in each domain; and the role of family and cultural context in shaping current communication. The term self-silencing was not introduced during interviews in order to avoid priming participants with the theoretical framework. Instead, questions focused on how participants expressed or withheld thoughts and feelings in specific relational contexts, allowing patterns related to self-silencing to emerge inductively. Second, 43 participants completed written reflections (500–1000 words). In contrast to the interviews, participants were provided with a brief description of self-silencing and explicitly invited to reflect on whether and how this pattern appeared in their own communication. This approach enabled participants to engage directly with the concept and assess its relevance to their own experiences. Several participants noted minimal self-silencing or explicitly rejected the construct, suggesting genuine reflection rather than uniform confirmation. The two sources differed in their conceptual prompting. In the interviews, the construct was described but not named, and questions remained open-ended; participants spontaneously raised relationships beyond romantic partners (notably friends and family) as well as cultural considerations, and follow-up questions adapted to the domains they introduced. In the written reflections, collected after the interviews, the construct was explicitly named as self-silencing, allowing participants to engage with the term itself. Both sources were analysed together as a single dataset and produced convergent patterns: participants across both described similar relational configurations of self-silencing, and no theme was supported exclusively by one data type.

### 2.3. Data Analysis

Interview transcripts and written reflections were analysed using reflexive thematic analysis (Braun & Clarke, 2006, 2019, 2021). Reflexive thematic analysis was chosen over coding reliability or codebook approaches because the study aimed to develop an interpretive account of how self-silencing operates across relational contexts rather than to apply a predetermined coding framework. The research questions asked how self-silencing varies within individuals rather than measuring its frequency, requiring an approach in which themes are generated through the researcher’s sustained engagement with the data (Braun & Clarke, 2021). Analysis followed six phases. During familiarisation, the author read all transcripts and reflections multiple times, noting initial observations about relational variation in expression and silence. Systematic coding was then conducted across the full dataset, generating initial codes that attended to how participants described their communication across different relationships, what they identified as shaping their expression or silence, and where their patterns varied or remained consistent. Codes were then organised into candidate themes through iterative comparison, with themes reviewed against both the coded extracts and the full dataset to ensure coherence and distinctiveness. To enable within-person comparison, each participant’s account was first analysed as a whole to identify whether and how self-silencing varied across the relationships they talked about, before cross-participant patterns were examined. Pseudonyms were assigned to all participants. In the Findings, each quoted excerpt is identified as drawn from an interview or written reflection. The author is a German cross-cultural researcher based in Thailand, whose prolonged immersion in Thai culture provides familiarity with Thai interpersonal norms and communication patterns. Interviews were conducted by the author and trained research assistants, whose involvement facilitated rapport and offered perspectives from within the culture that the author considered alongside her own interpretations. The author maintained a reflexive journal throughout the analysis. As a Western researcher working in a context where open self-expression is commonly regarded as psychologically healthy, she remained attentive to the risk of reading culturally valued restraint, such as kreng jai, as passivity or deference rather than as a considered way of showing care and respect.

## 3. Results

Self-silencing did not emerge as a stable, trait-like tendency applied uniformly across relationships. Instead, participants described silencing themselves in some relationships while expressing themselves freely in others. Four interconnected themes emerged from the data: self-silencing varied across relationships within the same person; what participants learned at home could be revised through later relationships; cultural values provided a way of explaining silence; and sustained suppression carried cumulative costs. The meaning of self-silencing differed by gender across these themes.

### 3.1. “It Depends Who I’m Talking to”

Across the dataset, self-silencing varied with participants’ expectations about how expression would be received. Participants spoke openly where they anticipated acceptance and self-silenced where they expected dismissal or conflict. Three configurations emerged.

#### 3.1.1. Safe at Home, Guarded Outside

Several participants referred to families that encouraged open expression, yet self-silenced in friendships or romantic relationships where acceptance felt uncertain. What distinguished these contexts was not a lack of communication skills but uncertainty about how expression would be received in a specific relationship. Joy provided a clear illustration. She traced her self-silencing not to her family but to a traumatic friendship in which her close friend “was aggressive” and would hit her if she did not comply. This experience left her feeling that she had “lost [her] confidence” and “didn’t know how to deal with close relationships … when we have conflict.” In contrast, she reported no self-silencing with her parents, recalled their relationship as “extremely stable” and grounded in “love and trust”: “When I stay with my parents… I can feel the love and trust … But with friends or boyfriends, I cannot confirm that I’m the most important to them.” (interview).

Across similar accounts, participants who had learned that expression was acceptable at home nevertheless withheld thoughts and feelings in relationships where acceptance felt less certain. Some suppressed emotions to manage how they were perceived: one participant avoided appearing “weak” or “emotional” in friendships despite growing up in a family that “fostered a spirit of debate,” while another withheld frustration from her romantic partner to avoid hurting him. Others defaulted to agreement, habitually going along with unwanted activities or choosing “silence or agreement,” reasoning that “it’s better to let it go” (reflection). One participant captured the split neatly: he could “be [himself]” with family but “acted another way” with friends (reflection). Chan highlights a different mechanism. Having rarely observed conflict between his parents, he lacked a model for handling disagreement and developed a strategy of suppressing negative feelings to avoid escalation: “I’m just disappointed, but I don’t want to say it … I don’t want to make it worse” (reflection). Whether driven by relational uncertainty or by the absence of a model for disagreement, self-silencing in these accounts was shaped by the specific relational context rather than by a general tendency toward silence.

#### 3.1.2. Finding Voice Beyond the Family

Other participants showed the reverse pattern: they self-silenced within the family but communicated openly with friends or romantic partners. Here hierarchy and emotional volatility within the family constrained expression, while egalitarian and responsive relationships outside it enabled openness. Nat recalled how disagreement with his parents was dismissed “due to my position in the family,” linking this to expectations of respect and *kreng jai*. As the oldest male, he was expected to be “the man of the house,” while his sister “was able to express her emotions more and had more freedom” (reflection), a gendered division that shaped both where and why he self-silenced. Yaya similarly spoke of parents with “hot tempers” who were “not very willing to listen,” leading her to remain silent at home. In contrast, her romantic relationship provided a different relational experience: “With him, I can clearly feel that I am loved … I feel relaxed and able to be myself” (reflection). Other participants reported similar tendencies, finding communication “easier and more open” (reflection) with friends than with family, where direct refusal felt inappropriate, or self-silencing at home while communicating directly with partners and colleagues. Throughout these experiences, self-silencing reflected not only past learning but ongoing relational feedback, and in some instances, relationships experienced as safe shifted participants’ communication more broadly.

#### 3.1.3. No Safe Harbour

A third group self-silenced across family, friendship, and romantic contexts, experiencing most relationships as conditional. In these accounts, participants had learned through modelling, hierarchy, or direct threat that expression was broadly unsafe, and silence functioned as a default strategy rather than a context-specific response. Nong traced this pattern to her position as the youngest child, learning that maintaining relationships required obedience: “I always obey … not only for my ex, my family, also for friend” (interview). Despite recognising that she was “consuming myself,” she felt unable to change (interview). Others spoke of strong modelling within the family. Several participants grew up observing parents who avoided conflict or suppressed disagreement, and each reproduced these patterns in their own relationships, becoming “people-pleasers” who suppressed their needs until they felt “constantly drained of energy” (reflection). Thanyapat’s account was particularly striking: her parents’ arguments would escalate until both reached their “emotional limit,” then end in mutual withdrawal, with the next day proceeding “as if nothing had happened” (reflection). She absorbed this dynamic and described disagreements triggering a fight-or-flight response, not a decision about whether to speak, but a bodily reaction that preceded any deliberate choice.

In other accounts, self-silencing was driven not by modelling but by fear. Fah, who grew up with a violent father, avoided expressing disagreement to prevent harm: “I just want peace” (interview). Nan also suppressed her needs due to fear of abandonment. Unlike the previous groups, these participants lacked a relational context in which they expected their voice to be accepted, leaving self-silencing as a generalised relational strategy.

### 3.2. Revisable Scripts

Family socialisation provided participants with initial expectations about expression and relationships, but these expectations did not determine adult communication. Participants’ patterns of self-silencing shifted in response to subsequent relational experiences, which could either reinforce or revise what had been learned at home.

#### 3.2.1. Relationships That Rewrote the Script

Several participants referred to trajectories in which later relationships actively revised earlier family-based expectations, suggesting that self-silencing patterns are not fixed by early experience but responsive to ongoing relational feedback. Pat’s account illustrates this clearly: although he had internalised the belief that expressing emotions leads to conflict, a supportive partner provided an alternative experience, and he gradually began to open up. Others reported more abrupt turning points. Thi’s change followed relationship breakdown: after two years of self-silencing produced what he spoke of as a “toxic dynamic,” he committed to “being myself and speaking up about my feelings” (reflection). Pu described a deliberate reversal after a breakup, realising that he needed to “put myself first” and beginning to “openly state my feelings … at the beginning” of interactions (reflection). Jay actively rejected the patterns of his violent family, stating that he tried “anything and everything … to not be like what I saw” (reflection), and went on to build a long-term relationship characterised by open communication.

These active change trajectories were more common among male participants. Thi, Pat, Pu, and Jay all recalled specific catalytic moments that shifted their self-silencing. Female participants less often referred to such explicit turning points, though change was not absent: Kate, who grew up in an authoritarian household where she “wasn’t allowed to disagree” (reflection), shared how a supportive romantic partner helped her shift from holding back to direct engagement with conflict. Sera set boundaries after recognising her pattern. This asymmetry may reflect gendered social reinforcement: women’s self-silencing tends to be validated as caring, reducing the impetus to change, while men’s self-silencing may conflict with expectations of assertiveness, creating dissonance that motivates revision. This revisability worked in both directions. Participants from expressive family environments nevertheless withheld thoughts and feelings in peer or romantic contexts where acceptance felt less secure. Sera described herself as generally “very direct” (reflection) but selectively silenced herself with a particular friend whose feelings she wanted to protect, linking this to her role as the eldest sibling after her parents’ divorce. Even when individuals have learned that expression is acceptable, perceived relational risk can override previously learned openness, just as secure relationships can support new ways of communicating.

#### 3.2.2. Compounding Influences

For some participants, self-silencing was reinforced because family expectations, cultural values, and social roles all pointed in the same direction. When multiple influences aligned, self-silencing became more stable and harder to change, representing the limit of the revisability observed elsewhere in the data. Ploy described receiving explicit instructions to “be female and be gentle” and not insist on her own opinion, messages that aligned with her observations of her parents’ relationship. Even when she held strong views, she “still just … compromised” to avoid being perceived as “aggressive” (interview). Pak’s experience was similar: his parents modelled silence, his cultural context emphasised *kreng jai*, and his role as eldest son positioned him as “head of the family” (reflection). He recognised he was repeating his father’s patterns:

“When there is a conflict and I choose to self-silence, I feel highly uncomfortable, tense, and suppressed during the situation. However, I force myself to endure it because I desperately want to avoid saying harsh words that would hurt my partner. The real danger is that when the same issues resurface, the suppressed feelings accumulate over time and eventually lead me to explode, like those intense emotional outbursts.”

Comparable patterns appeared across cultural contexts: Sae described Thai family hierarchy where “younger people were expected to adjust more” (reflection), Jin linked Korean norms of respect for elders to his tendency to “obey” (interview), Nip described how *kreng jai*, *jai yen*, and *bunkhun* (indebted goodness) overlapped, each reinforcing the expectation of silence. Despite his parents encouraging openness, Nip chose to stay silent to avoid disappointing them, though he later recognised what this cost his relationships (reflection). Dev’s account was different: although his parents favoured him over his sisters “just cause they were girls,” he self-silenced with peers, staying silent when cousins made “racist, sexist and homophobic jokes” because he feared becoming “a pariah” in his community (reflection). His experience shows that gendered advantage in one context does not preclude silencing in another, and that discriminatory behaviour from peers can sustain self-silencing even when family dynamics support expression. Self-silencing was most entrenched when family modelling, cultural values, and social roles all reinforced the same expectation.

### 3.3. A Vocabulary for Silence

A number of Thai and Thai-Chinese participants explicitly identified cultural values as connected to their self-silencing. However, the data suggest that these values functioned less as independent causes and more as a way of explaining, legitimising, and morally framing patterns that had other relational origins. Prae, for example, identified *kreng jai* and *jai yen* as part of her communication style, linking them to her family’s quiet conflict avoidance and to the gendered expectation that “girls should be considerate and thoughtful about other people’s feelings” (reflection). Nat explained how *kreng jai* and gendered expectations were intertwined in his account of self-silencing:

“I was raised to keep my feelings to myself. With *kreng jai* where I would be aware of others’ feelings as well as the social norm of men to keep their feelings to themselves, I avoided expressing my own negative emotions to avoid burdening others. (reflection)”

In these accounts, *kreng jai* provided a culturally recognised way of explaining and justifying self-silencing. Two patterns in the data challenge the interpretation of cultural values as primary causes. First, participants did not apply these values uniformly across relationships. Nat self-silenced with his parents, where *kreng jai* and hierarchy converged, but communicated more openly with his romantic partner. Similarly, Nip identified *kreng jai* as his “main driving influence” (reflection), yet was more direct with his partner than with his parents, applying the value selectively depending on relational context. If cultural values alone drove the behaviour, self-silencing would be expected across relationships rather than concentrated in hierarchical ones. Second, self-silencing persisted even when cultural values were explicitly rejected. Peem stated, “I hate *kreng jai* and *jai yen*. I feel like it’s limiting people’s freedom to express themselves,” yet still recalled going silent when angry, with suppressed emotions eventually overwhelming him. His continued self-silencing despite rejecting these values suggests that the behaviour was rooted in other processes, in his case, his family’s pattern of unresolved conflict and the gendered message that “as a man, you shouldn’t be crying, and have to be emotionally strong” (reflection).

Participants from non-Thai backgrounds identified structurally similar values. Priya described “respect for elders” and “maintaining family harmony” in the Indian context (reflection), while Kate referred to “saving face” and “southern hospitality” as comparable norms in a Western setting (reflection). These parallels suggest that culturally specific concepts such as *kreng jai* reflect broader relational norms rather than unique cultural drivers of behaviour. Cultural values, then, provide a language and moral framework that legitimises self-silencing and may lower the threshold for silence by framing it as considerate. But they did not on their own determine when or where self-silencing happens; what mattered in participants’ experiences was relational dynamics such as hierarchy, emotional safety, and anticipated responses.

### 3.4. The Cost of Keeping Quiet

Self-silencing took varied behavioural forms and carried cumulative costs. Some participants did not simply remain silent but actively constructed socially acceptable reasons for their behaviour. May told friends she was “not feeling well” instead of admitting she needed to study, because she “did not want to seem unsocial” (reflection). Nisa told her mother she “would think about it” rather than refusing an unwanted internship directly. Parich told his girlfriend a weekend trip “sounded ok” when he was exhausted, then spent the trip “mentally checked out” (reflection). In each case, self-silencing required active cognitive work, constructing plausible alternatives, managing the gap between what was said and what was felt, rather than simple omission. Not all self-silencing was driven by fear. Top suppressed his own study needs to support friends academically, positioning himself as the “academic last resort” (reflection), a form of role-based responsibility rather than relational threat. Laya weighed “what the consequences will be if I don’t speak … what I’ll lose by speaking, and who I’m talking to,” experiencing chosen silence as beneficial “for both myself and the other person” (reflection). Pom’s mother taught him to conceal emotions from outsiders: “never let anyone see through your emotions. When people can read you, they will see your weakness” (reflection), while encouraging openness within the family. His selective concealment served self-protection in public rather than relational accommodation in private, showing that behaviours that look like self-silencing on the surface can serve different purposes in different settings. Where self-silencing was involuntary rather than strategic, its costs accumulated over time. Jid shared how suppressed disagreements “appear deeply when a new argument starts” (reflection), intensifying present conflicts with unresolved prior tensions. Peem linked the involuntary quality of his silence to its eventual consequences:

“Currently, I have a trait that I think is bad for me and it is being quiet when I’m mad. Most of the time, when I’m mad, I feel like I don’t want to say anything because it is going to make the situation worse, but as I keep it in me, it’s like a balloon that keeps getting inflated until it bursts. Turns out, it’s actually worse than saying something. I feel like it’s not running away from the problem nor trying to solve them.”

Several participants reported that unvoiced frustration accumulated across relationships, in one instance contributing to a relationship breakdown after months of suppressed resentment. Physical and psychological symptoms were widely reported, including insomnia, headaches, tension, emotional exhaustion, anxiety, and somatic symptoms such as nausea. Participants described “sleepless nights, migraines, mood swings, and a poor appetite,” feeling they were “consuming myself,” and in one case using vaping to “relieve stress” (reflection), indicating spillover into health-risk behaviours. These costs appeared across both context-dependent and pervasive patterns, suggesting that repeated suppression carries similar consequences regardless of scope. Not all participants experienced self-silencing as costly. Ploy described accommodation as “a way to show my love and care” (interview), Ming viewed yielding as reciprocal, and Orm reported resolving disagreements through calm reasoning without lasting tension. These accounts raise the question of whether presenting silence as care reflects a genuinely low-cost strategy or a way of making ongoing suppression psychologically tolerable.

## 4. Discussion

The findings suggest that self-silencing operates as a relationship-specific process rather than a stable personal characteristic, with participants silencing selectively based on the perceived security of each relationship.

This study offers within-person qualitative evidence consistent with the proposition that self-silencing schemas are malleable cognitions that change depending on relational demands and threats (Jack et al., 2026, p. 3; see also Jack & Ali, 2010). Previous research examined context-sensitivity at the between-group level (Jack & Dill, 1992; Jack et al., 2026). Participants could identify which relationships felt safe enough for expression and which did not, with perceived conditionality as the key distinction. Whereas earlier studies compared different groups in different situations, participant accounts show that these assessments operate relationship by relationship within the same person. This is consistent with the attachment theory concept of the secure base (Bowlby, 1988) and with La Guardia et al.’s (2000) finding that attachment security varies substantially within the same person across relational partners.

Family socialisation influenced participants’ initial expectations about expression but did not determine their adult communication. Participants from expressive families still self-silenced in fragile relationships, while those from silencing families communicated openly with accepting partners. While Jack (1991) theorised self-silencing as relational schemas shaped by cultural and gender-role socialisation, the present data show that these schemas can shift in adulthood. Participants did not simply repeat patterns learned at home; relationships that offered consistent acceptance could revise those patterns, and relationships that did not could introduce silence in people who had previously expressed themselves freely. Self-silencing is therefore not only relationship-specific in any given moment, but open to change over time. That family socialisation influenced but did not determine adult communication aligns with Baldwin’s (1992) proposal that people hold different relational schemas for different relationships, and with Thompson et al.’s (2001) finding that perceived care from the current partner, rather than recalled parental care, was associated with self-silencing among women. Assor et al. (2004) found that parental conditional regard is associated with introjected internalisation, a sense of internal compulsion to behave in prescribed ways accompanied by resentment, a pattern closely resembling what several participants described.

The data also suggest that behaviours resembling self-silencing may serve different functions. Some participants described deliberate, context-specific decisions to withhold expression, weighing costs and benefits before choosing silence. Others described silence as involuntary, a pattern they recognised as harmful but felt unable to change. This distinction has implications for how self-silencing is assessed and treated: a global STSS score would not differentiate between strategic restraint and compulsive suppression, yet the two may carry different psychological costs and respond to different interventions. Future research could measure self-silencing at the level of specific relationships rather than as a global score, separating person-level from relationship-level variation.

Male and female participants referred to different trajectories of change. Male participants more often identified specific moments that shifted their self-silencing, while female participants less often reported such turning points. Ussher and Perz (2010) found that men viewed self-silencing as an aspect of masculine stoicism that could serve as both identity resource and emotional constraint, a tension that may push toward change when the relational costs become clear. This study offers accounts of how these gendered trajectories unfold in practice, with men describing active revision and women’s silence more often going unchallenged because it was socially reinforced as caring. The present data suggest that men’s and women’s self-silencing may reflect different motivations, consistent with previous observations that self-silencing serves different purposes across genders (Jack & Ali, 2010; Remen et al., 2002). Men in this sample framed self-silencing around emotional control and concealment of vulnerability, consistent with masculine stoicism (Ussher & Perz, 2010). Women more often framed it as relational care, a form of emotion work (Hochschild, 1983) in which managing one’s own expression serves the relationship. This finding has implications for measurement. The STSS was developed from depressed women’s narratives and grounded in feminine gender-role schemas, including a Care as Self-Sacrifice subscale (Jack & Dill, 1992). It may not fully capture male self-silencing rooted in masculine stoicism (Ussher & Perz, 2010), and the same scores may reflect different underlying processes.

The finding that the Thai values of *kreng jai* (consideration for others’ feelings) and *jai yen* (staying calm and composed) provided a framework for silence rather than independently causing it has implications for how cultural values are understood in relation to psychological constructs. No participant applied these values uniformly; those who described *kreng jai* as central still expressed themselves freely in safe relationships, and one participant who explicitly rejected both values continued to self-silence. These values inform how participants understand and justify their silence, but relational context is associated with where it occurs. Research on Thai communication patterns supports this: Knutson et al. (2003) found that Thai communication culture prioritises social harmony, and that Thai university students showaed higher rhetorical reflection, adapting one’s expressed position toward what others want to hear, than their American counterparts. Intachakra (2012) argued that Thai politeness is fundamentally motivated by the concept of the “heart” (*jai*). The present findings go further by showing that these cultural prescriptions are applied selectively based on relational context, not uniformly, a distinction that has not previously been examined in the self-silencing literature. Thai society is not culturally homogeneous. Regional, class, urban-rural, generational, and ethnic differences shape how values such as *kreng jai* and *jai yen* are understood and practised. The participants in this study, drawn from international universities where instruction is in English, represent a relatively privileged, cosmopolitan population within Thai society. How cultural values shape communication may differ among individuals with less education, fewer economic resources, or less exposure to international contexts. The interpretation advanced here should therefore not be read as a claim about Thai culture in general but about how young adults from this particular demographic context drew on cultural values to make sense of their relational behaviour.

This interpretation also has precedent in the cross-cultural self-silencing literature. Jack and Ali (2010) argued that the STSS achieves conceptual equivalence across cultures because its items are worded without specifying particular cultural standards, allowing respondents to interpret them through their own cultural frameworks (see also Jack et al., 2026). Ali and Toner (2001) found that Caribbean immigrant women in Canada reported higher self-silencing than those in the Caribbean, suggesting that context contributes to self-silencing beyond shared cultural values alone. The present data extend this from a between-culture to a within-culture observation: the same cultural value was applied selectively rather than uniformly, and participants from Indian, American, and Singaporean backgrounds identified equivalent values in their own cultures and applied them with the same context-dependency.

These findings have clinical implications. If self-silencing is relationship-specific rather than trait-like, then assessment should identify which relationships activate self-silencing and what makes those relationships feel unsafe for expression. Participants who self-silenced selectively could identify relationships in which they communicated openly, suggesting that these relationships may serve as a resource in therapeutic work. That supportive romantic partners helped several participants shift toward openness suggests that couple-based interventions may be particularly relevant. Context-specific administration of the STSS, asking the same individual about different relationships, could capture the relationship-level processes revealed in this study’s qualitative data.

The clinical stakes are particularly high when self-silencing co-occurs with intimate partner violence or coercive control (Inman & London, 2022). Several participants reported suppressing expression in relationships involving physical aggression, bullying, or controlling behaviour, and the resulting silence removed the capacity to name mistreatment, set boundaries, or seek support. Identifying whether self-silencing serves relational accommodation or masks exposure to harmful behaviour is therefore a critical clinical distinction.

For practitioners working in Thai or other Southeast Asian contexts, the finding that *kreng jai* provides a way of framing silence rather than causing it suggests that cultural values need not be challenged directly. Rather, interventions can work within cultural frameworks by helping individuals distinguish between chosen accommodation and involuntary self-suppression. The concept of *jai yen* need not be framed as pathological; clinicians can help clients identify when calm communication serves their interests and when it masks genuine distress.

### Limitations and Directions for Future Research

Several limitations should be noted. The sample of 53 participants was drawn from international universities in Thailand where instruction is conducted in English, and participants came from relatively privileged backgrounds. While the cultural diversity of the sample strengthened the analysis by revealing that self-silencing patterns appeared across cultural groups, the sample was not designed for systematic cultural comparison, and the cultural values analysis draws most heavily on the accounts of Thai and Thai-Chinese participants. The written reflection component provided participants with a brief description of self-silencing, which may have directed attention toward the construct. However, several participants referred to minimal self-silencing or explicitly rejected it, and the interview component did not use the term, reducing concerns about demand characteristics. The retrospective nature of the data means that accounts of family origins may reflect current interpretations rather than accurately capturing childhood experiences, and because data were collected at a single time point, the study cannot address how self-silencing patterns develop or change over time. All participants were in emerging adulthood (Arnett, 2000), a stage characterised by identity exploration and relatively early-stage romantic relationships. Self-silencing patterns may differ among older adults or in longer-term partnerships where relational dynamics have had more time to stabilise.

These findings point to several directions for future research. Qualitative longitudinal studies following individuals through relational transitions could clarify how self-silencing patterns shift over time and whether change in one relationship transfers to others. Research with older adults or individuals in long-term partnerships would extend these findings beyond emerging adulthood. The within-person variation observed here also has implications for measurement: the STSS asks about behaviour in “close relationships” without specifying which relationship, and a global score may obscure meaningful variation. Self-silencing in same-sex romantic relationships, which may involve different relational dynamics, also warrants investigation. Future research examining self-silencing in Thai or other collectivist populations should attend to the distinction between strategic public self-presentation and relational self-suppression, behaviours that may look identical on the surface but serve different psychological functions.

## 5. Conclusions

This study explored context-dependent self-silencing at the within-person level, examining a theoretical proposition that Jack (1991) articulated over three decades ago but that the field’s reliance on between-group, single-context designs has left largely unexamined. Participants did not self-silence uniformly. They silenced selectively, based on the perceived security, conditionality, and power dynamics of each specific relationship. Family socialisation shaped how participants approached expression, but these patterns were revisable through concrete relational experiences that either confirmed or challenged earlier expectations. Thai values of *kreng jai* and *jai yen* gave participants a way to explain and justify their silence, though where silence occurred depended on relational context. The meaning of self-silencing differed by gender but not the pattern itself. These findings suggest that self-silencing research may benefit from attending not only to who self-silences, but to when, where, and in which relationships individuals choose silence over expression. The selective, relationship-specific nature of self-silencing connects to face negotiation theory (Ting-Toomey, 1988), which examines how individuals manage conflict differently depending on relational and cultural context. Future work could explore how self-silencing relates to face-saving strategies across collectivist and individualist settings.

## Data Availability

The data presented in this study are available on request from the corresponding author. The data are not publicly available due to privacy and confidentiality concerns, as participants were assured that their responses would remain confidential.
